# Strategies to Stay Alive: Adaptive Toolboxes for Living Well with Suicidal Behavior

**DOI:** 10.3390/ijerph18158013

**Published:** 2021-07-29

**Authors:** Bonnie Scarth, Jesse M. Bering, Ian Marsh, Vilma Santiago-Irizarry, Karl Andriessen

**Affiliations:** 1 Primary Mental Health, Suicide Prevention and Postvention, WellSouth Primary Health Network, Dunedin 9016, New Zealand; 2 Centre for Science Communication, University of Otago, Dunedin 9054, New Zealand; jesse.bering@otago.ac.nz; 3 School of Allied and Public Health Professions, Faculty of Medicine, Health and Social Care, Canterbury Christ Church University, Canterbury CT1 1QU, UK; ian.marsh@canterbury.ac.uk; 4 Department of Anthropology, Cornell University, Ithaca, NY 14853, USA; vs23@cornell.edu; 5 Centre for Mental Health, Melbourne School of Population and Global Health, The University of Melbourne, Melbourne, VIC 3010, Australia; karl.andriessen@unimelb.edu.au

**Keywords:** suicide, qualitative research, suicide prevention, lived experience, suicidal behavior

## Abstract

Suicidal behavior constitutes a major global problem. Qualitative research utilizing the first-hand experiences of those who have survived attempts to take their own lives can offer much in the way of understanding how to live well despite ongoing suicidal behavior. Given that suicidal intentions and behaviors occur within the person’s subjective construal, the solutions to living—and preferably living well—despite such inclinations must also be subjective and adaptive. The aim of this study was therefore to understand how individuals live with different aspects of their suicidal behavior and their use of effective strategies to protect themselves from future attempts. Thematic analysis of semi-structured, qualitative interviews with 17 participants with lived experience of suicidal behavior from the USA yielded two main themes: (i) the ‘dynamic relationship with suicidal behavior: living with, and through’, and (ii) ‘the toolbox’. Each of these themes had four subthemes. Participants in this study offered important insights into what helped them not just survive ongoing suicidal behavior, but how they created unique toolboxes to continue living, and to live well. These toolboxes contained personalized solutions to dealing with recurring threats to their subjective wellbeing and included diverse solutions from spirituality, pets, peer-support, participating in the arts, through to traditional therapeutic supports. Some participants also discussed the importance of broader social policy and societal changes that help them live. The findings highlight crucial implications for suicide prevention efforts, especially in terms of encouraging collaborations with the lived experience community and furthering a strengths-based approach to mitigating suicidal behaviors. We encourage the clinical community to work in partnership with service-users to enable them to generate effective solutions to living—and living well—through suicidal behavior.

## 1. Introduction

According to the World Health Organization (WHO), every year over 700,000 people die by suicide around the world, making it the 17PthP leading cause of death. Furthermore, community surveys undertaken in 21 countries by the WHO established the 12-month prevalence of suicidal ideation at approximately two percent [1] and the lifetime prevalence at nine percent [2]. Many people live with suicidal behavior (‘a spectrum of activities related to thoughts and behaviors that include suicidal thinking, suicide attempts, and suicide’) [3]) over a long period of time, sometimes a lifetime. The WHO surveys estimated that for those with a lifetime history of suicidal ideation, the probability of ever making a suicide plan was around 33 percent, and of ever attempting suicide approximately 30 percent [2].

Over recent decades, substantial progress has been made in the research on suicidal behavior, especially regarding epidemiological aspects, risk factors and population-based preventive approaches [4]. However, the evidence regarding the effectiveness of suicide prevention strategies is still limited [5]. Suicide prevention is defined by the Suicide Prevention Resource Centre as ‘activities implemented prior to the onset of an adverse health outcome (e.g., dying by suicide) and designed to reduce the potential that the adverse health outcome will take place’ [3]. Suicide prevention, therefore, aims to prevent death by suicide, and non-fatal suicidal behavior is a key indication that someone is at-risk of death by suicide.

Given the large number of people who experience suicidal behavior [6], it is somewhat surprising that only a few studies have focused on first-hand accounts of what it is like to live with and through suicidal behavior, and particularly to live well (subjectively speaking) [7]. Although suicide prevention is typically associated with the triaging of acute psychological distress in a person immediately at risk of taking their own life, resources must also be devoted to helping the ‘chronically suicidal’ live with such recurring, even lifelong, behavior. To prevent suicide, however, we must first understand it.

According to Shneidman [8] “…our best route to understanding suicide is not through the study of the structure of the brain, nor the study of social statistics, nor the study of mental diseases, but directly through the study of human emotions described in plain English, in the words of the suicidal person. (p.24)” Qualitative research enables us to analyze the words and emotions of people who have experienced suicide, to better understand it.

White (2016) [9] comprehensively reviewed the qualitative literature regarding suicidal ideation and attempts, and noted that most research focused on the perspectives of either professionals or non-suicidal people [9]. However, a review by Lakeman and Fitzgerald (2008) [7] found 12 qualitative studies that included accounts from people who had survived suicidal behavior. The authors found several connected themes related to the experience of suffering and struggle, in addition to themes on the importance of finding connection [7]. The turning point for people experiencing suicidal behavior usually happened quite suddenly and tended to occur after the right kind of connection (interpersonal or spiritual) was made [7]. Furthermore, Lakeman and Fitzgerald noted that suicide was perceived by participants in these studies as being simultaneously a way to cope and as a sign of personal failure [7].

Quantitative studies cannot capture the phenomenological experience of ‘being’ suicidal in this way, and therefore first-person accounts of such individuals can provide invaluable insights that contribute to our understanding of suicidal behavior and its prevention [8]. Given the shortage of research regarding what it is like to live with suicidal behavior over a long period of time, the present study aimed to explore the personal experiences of those living with suicidal behavior, and the strategies that people with suicidal ideation or attempts adopt to cope with these significant challenges. Furthermore, we examine how these participants live, and often live well, with suicidal behavior and how learning from their experience may aid suicide prevention efforts.

## 2. Materials and Methods

The study is based on data collected from an ethnographic and emergent qualitative study carried out in the USA between November 2015 and June 2017 by the first author (BS). The study was funded by a Fulbright scholarship, Cornell University fees scholarship, and Lois Roth Endowment and initially focused on public and expert perceptions and understandings of suicide prevention/intervention/postvention strategies for youth suicide. However, the participants included in this paper are those who self-disclosed personal suicidal behavior, with most experiencing suicidal behavior since they were very young (often as children or at least adolescents). Hence, although they participated in a study on youth suicide, their narratives spanned their life course, including how they managed their ongoing suicidal behaviors and recovery at different time points. Hence, this paper focused on how people experience living with suicidal behavior and their key strategies to manage and live well with such tendencies. 

JMB incorporated some data excerpts from these research interviews into a book he authored on suicide [10] and BS has delivered workshops and conference presentations from the data [11,12,13].

### 2.1. Ethics Approval

Ethical approval was granted by Cornell University’s Institutional Review Board (protocol number 1509005808) on 10 December 2015.

### 2.2. Interviewees

The original study included 24 participants. However, for purposes of the present study, only data from those who spontaneously self-disclosed having engaged in one or more suicide attempts and/or having been through a significant period of suicidal ideation, as well as discussed what had helped them to live/live well despite this history, were included in the analyses. Table 1 presents the sociodemographic characteristics of the interviewees (*N* = 17).

### 2.3. Procedure and Data Collection

Interviews were open-ended or what is known as narrative interviews [14], and because the research was ethnographic, open-ended and emergent, a topic-guide was not required [15]. With this approach, the researchers decide on the topic they wish to investigate, but then allow the participants to direct the narrative and decide what they feel is important to the topic, as opposed to the researcher deciding [14]. This allows the participants to maintain some level of control over what they feel comfortable including or excluding from their narrative. The open-ended approach also allows for unexpected or innovative findings that the researcher may not have thought of or considered in relation to their topic [16].

Regarding the self-disclosures of suicidal behavior, those participants who did disclose usually did so spontaneously at the beginning of the interview in response to the researcher’s standard opening question that asked why they chose to participate in the research. A small minority of participants (*n* = 2) self-disclosed later in the interview, after the researcher had asked a clarifying question as to whether they had personal experience of suicide (such as bereavement, professional work with suicide, etc.). The researcher did not consistently ask this question; rather, it was asked only if the participant was discussing an aspect of suicide in such a way as to indicate that they may have personal experience. If the interviewee self-disclosed personal experience of suicidal behavior, the researcher asked all such participants what helped them live with or manage their suicidal behavior. Thus, this article is based largely on responses to that question.

Participants aged 18 years and older were recruited via snowballing (word-of-mouth), fliers, and social media (including email listservs). The advertisements and participant information sheet specified that the research questions would pertain to youth suicide prevention. Advertisements advised that potential participants who had experienced suicidal behavior in the past 12 months should consider not taking part due to the emotional risk, but this was not an exclusion criterion, rather it was general advice. A sheet of free help services was provided, and the interviewer (BS) offered to follow up with a check-in phone call or text to participants at a time that suited after the interview.

Participants provided written informed consent prior to the interview, after the interviewer had checked that they had read and understood the study information and consent form.

All but two of the interviews were in person (at a location chosen by the participant), with one conducted by Skype and one by telephone. The IRB allowed for interviews to be conducted by phone or video chat. For in-person interviews, BS provided a non-alcoholic drink of the participant’s choice and biscuits or fruit, depending on the participants’ preference.

All interviews were audio-recorded (with signed permission) except one in-person interview in which the participant did not want to be recorded, so the researcher took field notes for that interview instead. The interview lengths ranged from 40 min to almost 4 h, with the average interview time around 90 min. A transcription service was employed to transcribe the interviews verbatim (after signing a confidentiality agreement). Excerpts in this paper have been edited to remove descriptions of suicide methods and details that risk identifying interviewees.

The interviewer (BS) had 10 years of research-interviewing experience on sensitive topics such as bereavement, grief, trauma, addiction, and terminal illness, in addition to extensive professional experience working in social services such as family violence, sexual health, and parenting services. VSI and JMB supervised the research project and regular supervision meetings were held to discuss progress and minimize researcher bias.

### 2.4. Data Analysis

Thematic analysis is a method for organizing qualitative data through identifying patterns (themes) within and across data [17]. Researcher BS followed Braun and Clarke’s (2006) [17] six iterative steps of thematic analysis. This involved: (1) transcribing the recordings and/or repeated reading of the data to familiarize oneself with the data; (2) generating initial codes; (3) searching for initial themes; (4) reviewing themes against the data; (5) defining and naming themes, and; (6) producing the report. The analysis adopted an inductive approach, involving “bottom up” coding and analysis. Such a data-driven analysis yields themes that do not mirror the interview questions or the researchers’ interest in the topic [17].

All interviews were coded twice line by line and paragraph by paragraph on MAXQDA software. While coding organizes data into ideas and then groups of ideas [17], these groups are not the themes per se. BS used a latent form of thematic analysis, whereby she endeavored to reflexively examine the underlying assumptions and ideas that were forming the data, which fits with the use of inductive coding. A latent analysis involves interpretive work [17] and BS wrote interpretive memos as she coded to aid this process.

When deciding on the themes, BS would draw together several groups of codes (often seemingly contradictory yet related codes) and interrogate what she believed their latent meanings might be. For example, she would reflexively consider (using her own sensitization from personal and professional experience related to the excerpts, as well as extensive reading) the specific words people used to describe their experiences, or their particular framing or understanding of their suicidal experience (e.g., using neurological or medical terminology, or using cultural or religious understandings of their experience). The researchers, especially BS and KA when writing up the key themes in this paper, held regular team meetings to minimize researcher bias.

A lay report was sent to participants by email on 21 February 2018, which primarily focused on summarizing the data analysis in terms of what helped people decide to live, despite ongoing suicidal behavior. The two key themes in this article are derived from the analysis discussed in lay terms in that report.

## 3. Results

The analysis yielded two key themes, each with four subthemes (Table 2). In what follows, names of participants are pseudonyms. Edits in participant quotes are indicated by (…) and all references to specific suicide methods, medication brands, locations and other potentially identifying information have been edited out. 

### 3.1. Theme 1: The Dynamic Relationship with Suicidal Behavior: Living with, and Through

Participants described their ongoing relationship with suicidal behavior as dynamic, with four subthemes described across the data: fighting for life, ambivalence, acceptance, and finding/holding hope. Most participants described moving through each of these states over their lifetime, though some moved in and out of them more regularly, or experienced different states simultaneously, as some excerpts demonstrate. However, some participants stayed more settled in one particular state. The following section describes each subtheme or relationship with suicidal behavior in more detail.

#### 3.1.1. Fighting for Life

‘Fighting for Life’ refers to participants’ descriptions of being in an acute state of managing suicidal behavior, with ongoing, intense and persistent thoughts of suicide, whereby they had to ‘fight’ to survive. Most participants described the process of managing and battling persistent thoughts of suicide as “exhausting” in some form. Their exhaustion was a result of constant, intrusive thoughts of suicide and the energy it took to manage the thoughts to ensure that they did not act on them.

These participants still had to continue with their day-to-day life while managing intense periods of suicidal behavior, with responsibilities such as parenting, work, and relationships. Participants also described how much of their exhaustion during these periods was due to having to keep up a pretense of being “normal” or at least “okay”. This perceived necessary pretense tended to make their suicidal behavior more intense. On the other hand, those who had understanding workplaces or worked in peer-support settings found these periods of suicidal behavior more manageable, as they were able to be honest and get the support they needed. For example:

*In every case when I would reach out, and when I would be honest I always got help. Sometimes it is just the act of being honest and it is not really the help, for me, a lot of the time it is just not hiding out, not keeping secrets*.

(Lucas)

Nevertheless, the fighting for life stage required constant vigilance and a unique set of tools. While this period of suicidal behavior was all-consuming and intense for participants, it also illustrated how self-aware they were of their process and what they needed to manage to stay alive:

*It turns in on myself and I see things as a way to kill myself, like things that don’t normally look like ways to kill myself become ways to kill myself… it is a constant thought.… [M]y clues to me that I need to go to the hospital are when I start planning out how to get rid of my stuff …one of my primary reasons for living is I want to live with my cats, and I don’t want my cats to go to other houses because I know they would be split up. By the time I don’t care about my cats, and I don’t care about whether or not killing myself would hurt my best friend’s family, and the most important thing is when I can no longer keep myself safe, and that is when I can’t drive, I can’t walk by buses, I can’t look at scissors … I become consumed with thoughts about how to die. I become consumed with thoughts about how to prepare my things for when I am gone, and that is when I go to the hospital….*

(Candace)

While eleven of the participants had made at least one suicide attempt (usually multiple) over their lifetime, many lived through these periods of intense suicidal behavior having never made a suicide attempt. Some of these participants tried to actively prevent their own suicide while feeling suicidal.

*I remember it was strongest when I was in high school—maybe there were times before that—but I think maybe earlier…having periods where I thought, “I want to kill myself. I want to die. What can I do to prevent that?”*

(Willow)

A detailed and reliable analysis on the differences between those participants who made suicide attempts during this intense period of suicidal behavior, and those who did not, is beyond the scope of this paper. However, broadly speaking, restricted access to means and appropriate support appeared to be key factors behind whether or not participants experienced the ‘fighting for life’ period without making a suicide attempt.

#### 3.1.2. Ambivalence

The ‘ambivalence’ subtheme describes an aspect of suicidal behavior in which participants described feeling neither acutely suicidal nor invested in staying alive. Ambivalence was associated with ongoing “back of the mind” suicidal ideation or non-active suicide attempts, and was not associated with the exhaustion of ‘fighting for life’. Whereas participants described actively looking for ways to take their own life in the ‘fighting for life’ theme, and often described suicide attempts during these periods, their thoughts of dying in the ambivalence theme were not only less tiring but sometimes even described as pleasant or helpful.

*I remember laying there just wishing, like just thinking, “Wouldn’t it be nice to just …and put your head down on this pillow and just let it go? Just resign to that?”…It is really tempting. It is almost romantic, like it sort of calls to you like wouldn’t that be the sweetest thing?*

(Alice)

In cases where a suicide attempt during the ambivalence stage, participants tended to reflect that it was ‘not serious’, that it was a ‘game’, or a gamble with death.

*I don’t know why I did it; I think I did it mostly for fun…I don’t know how serious those (suicide attempts) were really—I really can’t tell you. I wouldn’t’ have objected to however it turned out, I don’t think, but it was kind of a game at that point … in trying to sort of snatch victory from the jaws of defeat things worked out quite well…I don’t believe anybody who has ever tried to commit suicide is safe. I don’t. The only thing that has happened to me in that regard is … I am increasingly less frightened of death. I am not real enthusiastic about dying, especially if it is long and painful but I am not afraid of death at all.*

(Lucas)

For some participants, the ambivalence came after the suicide of a close loved one.

*In any case, something was available, I was curious and I never quite dealt with my grief very well and my mind sort of went, “Well what was he thinking?” and so I just sort of repeated it. It wasn’t that I was unhappy, it wasn’t that I wanted to … it was just, “What do you think when you do it?” Stupid! [laughs].*

(Pippa)

As illustrated by these excerpts, ambivalence does not indicate less risk than ‘fighting for life’, but subjectively, it is a less exhausting and all-consuming period of suicidal behavior to live with.

#### 3.1.3. Acceptance

The ‘acceptance’ phase of suicidal behavior refers to participants who described coming to the point of accepting that they would likely always have to live with some form of suicidal behavior. This acceptance actually formed a part of their self-directed toolbox, as will be discussed later, as those participants who came to accept suicide as probably being a constant in their lives, tended to find it more manageable.

*The reality is, in my life…it is likely that I am going to have another bad time. … I am not going to be happy about it, but I have to understand, just like I can’t walk and no matter how much I want to walk it is not going to happen, and so there is a similar—and not in as completely an obvious sense—in which I just have to be ready for it (suicidal behavior) to be a problem…*

(Ludo)

Some participants also pointed to the importance of spirituality, which was a key part of their self-directed toolbox and played a role in the acceptance of suicidal behavior as an ongoing part of their lives, but not something to act upon.

*Here and now is more my concern, and the concern is the more here and the more now the better. That is a big AA teaching…It (suicidal ideation) is not overwhelming, it is just always present… I think that suicide is a sin in a sort of conventional sense because you are taking control out of the hands … like a spiritual authority, from your body, whatever, you are taking it away … So I am in sort of this peculiar spot right now. I think it is a good spot, actually, where I am not really … I am not sort of as goal-directed and I think that is good. I am glad about that.*

(Lucas)

#### 3.1.4. Finding and Holding Hope

‘Finding or holding hope’ describes the point where participants had moved—at least for a time—beyond fighting for their life, ambivalence, and acceptance, and found hope to hold on to and live for. This was not to say that they did not simultaneously believe that they may one day feel suicidal or make a suicide attempt again. Some continued to live with almost daily suicidal ideation to some degree, while still holding hope. Participants sought hope through a multitude of avenues, which made up their particular toolbox for living through or with suicidal behavior. It is important to note, however, that those participants who found the acceptance stage to be most helpful in living, and did not experience hope, did not see hope as necessary or even helpful in living well. However, for some participants, hope was an important point to reach—finding something that gave them hope in themselves or the world at large.

*I think being close to people in general…To me, yes, I do think that the thing that is keeping us sort of here, keeping us hopeful about the world in general is meaningful relationships with people.*

(Hayden)

Perhaps counterintuitively, it was those participants who had been through repeated stages of acute suicidal behavior—the fighting for life stage—and made multiple suicide attempts who were more likely to reach the holding hope stage. This was often due to the necessity to consciously examine one’s life and recognize that they had lived through suicidal periods before, or survived a suicide attempt and were able to find hope and happiness again.

*It is something that with a little bit of work you could get through, yeah, the pain is bad but it hasn’t always been like that. There were good times and there can be good times, you know? It is to try and focus on how to get them back, rather than how to make the pain end, is how to make joy possible again—because it is, you just don’t see it at the moment.*

(Alan)

### 3.2. Theme 2: The Toolbox

The ‘Toolbox’ theme describes the multitude of coping tools that participants utilized when managing different stages of suicidal behavior. Different stages of suicidal behavior required different toolboxes. The participants had become adept at understanding what they needed to keep themselves alive at different stages of suicidal behavior, and were resourceful in drawing on their unique set of circumstances. 

#### 3.2.1. The Self-Directed/Discovered Toolbox

The ‘Self-Directed/Discovered’ toolbox describes all the tools that participants had found and utilized in their everyday lives; it was the most comprehensive toolbox. The ‘self-directed’ component refers to the fact that participants largely used these tools in a self-managing way, though often in partnership with a therapist or peer-supporter. The ‘discovered’ aspect pertains to how these participants had found, whether intentionally or by chance, each of these tools in their day-to-day lives or environment.

The components in this toolbox enhanced participants’ lives in such a way that made their ‘lives worth living’ [18] when they may otherwise have given up. As such, the self-directed toolbox was most useful when participants were at the ambivalent, finding hope, or acceptance stages of suicidal behavior. As one participant notes in the excerpt from the aforementioned fighting for life section, her cats, which were a part of her self-directed toolbox, were no longer enough to help her manage her suicidal behavior once she was at the acute stage. They were, however, a useful signal for when she needed extra help via psychiatric care, which was in her ‘conventional’ toolbox.

Tools in the self-directed/discovered toolbox that participants consistently mentioned across the data, included: exercise, nature, meditation, spirituality, social connection, music, art, other suicide survivor’s stories of recovery, and, perhaps counter-intuitively, some participants found the thought of suicide itself to be helpful in keeping them alive.

*Some of it (suicidal ideation) was…empowering … because I was so unhappy and I couldn’t do anything about it, and no one would help me. It was like, “Okay, I know I can always do this. I have this option, so if it hurts too much …” you ultimately have the last word and there always is something you can do if it gets too bad, and that is comforting.*

(Alice)

Other key parts to this toolbox included spending time in nature, which often overlapped with the tools of exercise, spirituality, meditation, and pets. However, a couple of participants also mentioned making a suicide attempt or plan after the death of a much-loved pet, so while pets offered some protection, they also became a risk factor. Overall, however, these tools brought a sense of calm and grounding to participants’ lives.

*I’m lucky because I have a little dog and I have to take him for walks and that usually helps, but I still have my bad days and bad nights, but I think I am at a good point where I am not going to kill myself.*

(Janine)

*I had a chance to spend a few years spending a lot of time by myself in nature, time alone in nature, physical exercise… I realized something had changed in me because the old pathways that would go to this place where I was just like, [having] an uncontrollable self-destructive urge constantly alighting on my brain every day was gone. I didn’t go there, and I was really unhappy, but I didn’t go there…But for me, you know, that has to do with quiet. Meditation was also something…I started studying Buddhism, and I would meditate …Yes, meditation outside, walking outside, running outside.*

(Jeff)

Almost all participants had fostered spiritual beliefs of some kind that they found useful in helping them live through or with suicidal behavior, with many attending a church because they enjoyed the community support. Other participants deliberately chose a belief system—such as karma or reincarnation—that would help deter them from suicide.

*I think…what saved me from taking any steps, was also the karmic thought in my mind saying, “Oh, this all seems really familiar. I bet I have done this before in other lifetimes,” and it kind of feels like I did it before and then realize that that was not the right thing to do, and if I do it again I am just going to be repeating the cycle. Okay, maybe I need to hang on and figure out a way to get out of it.*

(Willow)

*I don’t think being an atheist would be very helpful to me … For me what faith or whatever that I have is a function that I just live better that way. When I lose faith then I become more dangerous to myself.*

(Lucas)

Storytelling was another tool repeatedly mentioned across interviews, and like the arts (e.g., theatre, painting, music, and sculpture were all mentioned as healing), it provided a vehicle for both expressing one’s own suffering and achieving catharsis, as well as learning from other people’s stories and identifying similarities, feeling less alone, and finding hope.

*Telling your story is clearly a therapeutic kind of thing. Hearing other people with similar kinds of symptoms, and how it works out for them, and their stories is very powerful.*

(Martin)

As a tool for managing or recovering from suicidal behavior, storytelling intersected with peer-support, both for those participants who worked or volunteered as peer-supporters (reported by four participants in this study), and for those who utilized peer-support services or groups (almost all had at some point, though many in an informal capacity).

*For a long time I was like, “I will just keep this to myself,” but being a certified peer specialist you have to become comfortable talking about your own experience, and so for two weeks that is all we did—talk about our experiences. I remember there was one man and he was like, “I have these random depressions. They show up every five years, I want to kill myself and then I am fine,” and then he is like, “And five years later it shows up again,” and I was like, “Me too!” He was like, “I have never met someone with that type of experience,” and I was like, “Neither have I!”*

(Jane)

Social connection, whether through friendship or family, was repeatedly cited as a key tool for participants, and as previously noted, was an important part of holding/finding hope.

*I think it was helpful that I had a few very dear friends who, like, beg is not the right word, but would talk me out of it… when I look back at it I am shocked that I am still alive because…I didn’t trust a lot of people around me. I would say in a very big way I owe my life to this one friend and my cousin who sort of talked me through a lot of things… I think that I am exceptionally lucky in that I had a few friends who looked out for me very much, and I have an extended family that still likes me, despite the fact that we disagree on a few things.*

(Angela)

Children were mentioned several times by participants as a motivation to keep living, despite managing suicidal behavior.

*But of course I couldn’t think about really killing myself at that point because I couldn’t do that to my kids…So that stopped me from going that far in the fantasies, like, “Well they need me and I would traumatize them if they came in the room and I was dead, so I can’t do that…. So I needed to make sure that I didn’t commit suicide.*

(Willow)

#### 3.2.2. The Conventional/Medical Toolbox

The ‘conventional/medical’ toolbox describes those tools that participants primarily utilized for managing acute suicidal behavior and included medication, psychiatric care, therapy, helplines, and peer support groups and services. Most of the components of this toolbox were only mentioned in the context of acute suicidal behavior. However, while peer support and therapy were usually initiated during an acute stage of suicidal ideation or after a suicide attempt, they tended to be sustained long-term as a prevention mechanism. Finding the right fit with a therapist and/or medication often took time, and while a particular therapist or medication may help for a period, participants often found they had to change medication or therapy as their needs evolved.

While a number of participants described psychiatric care as unhelpful, several participants cited in-patient stays in psychiatric care as beneficial when they were acutely suicidal. However, the experienced benefit of the stay tended to be due to either the therapeutic relationships or the connections they formed with other patients.

*So, I was over there (psychiatric unit) for an entire month … and the psychiatrist that worked with me…I got along with him real well and so that was really beneficial. We had some nice discussions…I finally got out after a month and there was a transfer of my medication out to…and I started seeing a therapist out there as well.*

(Eric)

Many participants discussed particular therapeutic modalities such as Cognitive Behavior Therapy (CBT) and Dialectical Behavior Therapy (DBT) that helped, with DBT mentioned most frequently.

*Because one of the things I learned most from DBT is I need to think of me, not as a victim but a survivor.*

(Sally)

Almost all participants described the benefits of peer-support and being with others in a group who had similar experiences. The key benefit was finding empathy and understanding without judgment.

*If I am in some sort of peer support setting, whether it is individual or in a group, or whatever it is, I know that person gets it and it is so much less scary to tell those people… that you really do connect with when you go to a hospital… you connect to other people in the program and those are the biggest influences on you. If you are in a group with a good set of people who are active and participating, and like encouraging in the group you are going to get a lot more out of it, and it doesn’t matter who is standing at the front of that room, it is about the group you are surrounded by.*

(Jane)

Most participants had been on psychiatric medication at some point, and most were still taking a combination of medication that would change from time to time. These participants expected to be on medication for the rest of their lives, even if they were not particularly happy about it. While many participants described difficulties, such as negative side-effects or withdrawals after cessation, some also found it helpful.

*I started seeing a counsellor when I was thirteen, I think—in those early teen years. And then it has been an ongoing sort of come and go… And then just a couple of months ago… it got to a breaking point and I started taking medication. I feel better. I started taking… and it is really good.*

(Alice)

Some participants found that just having the medication, even if they did not take it, was helpful.

*I don’t take the antidepressants now, I don’t like the side effects. But I still carry (anti-anxiety medication) with me. It helps to know I have it.*

(Meredith)

#### 3.2.3. The Life Circumstances Toolbox

The ‘life circumstances’ toolbox refers to the circumstantial tools participants drew on that were naturally a part of their lives, and could be consciously utilized and enhanced to help them live through or with their suicidal behavior. While there are similarities with the self-directed toolbox, in that participants also consciously found and self-directed when engaging this toolbox, ‘life circumstances’ was more ‘big picture’, societal circumstances, rather than the day-to-day. As such, it was not a toolbox that they could actively employ for functioning in their day-to-day lives, such as having a dog to walk or going for a daily run, but rather one that included circumstances that they could reflect on to motivate them to live through a period of suicidal behavior.

Examples of tools in this toolbox included the legalization of same-sex marriage and campaigns such as ‘It Gets Better’ [19] for LGBTQ+ youth, or media projects against stigma and discrimination surrounding mental illness and suicide, as well as individualized circumstances, such as the ability to relocate and reinvent oneself or connecting with one’s identity.

Some participants identified as LGBTQ+ and had struggled with suicidal behavior as a result of a non-accepting and often bullying community. A few mentioned the ‘It Gets Better’ [19] campaign and the knowledge that they could one day relocate to a more inclusive place. This idea relates to the holding hope stage of suicidal behavior.

*For a long time, I was like, “When I am an adult I can travel and work in theatre and do all these things,” and that was really helpful to me.*

(Angela)

Government policy changes in mental health and suicide prevention were mentioned as making positive or negative differences to participants’ wellbeing. For example, bridge barriers were discussed as having a positive impact and demonstrating care.

*To me they (bridge barriers) sent the message of…we care enough now to say, “Don’t jump off the bridges. We don’t want you to kill yourself.” It is the opposite of what having no fences said: “Oh, we don’t care, if you jump off, you jump off and we will just weed you out.” Weed out the weak.*

(Duane)

*The bridges (barriers) are about “the community cares”. That is what the message is.*

(Pippa)

Outside of specific suicide and mental health policy, other initiatives such as same-sex marriage, which created a more equal and inclusive society, made positive differences for participants.

*It will be thirty years (together) this year. Yeah, we got legally married as soon as it was legal…Anyway it is quite an amazing … I specifically call him my ‘husband’ now, and I used to call him my ‘partner’ or my, you know, my ‘boyfriend’. But now I specifically call him my husband because there is something about having … of course depression and, you know, the sense of alienation has to do with how much you feel a part of the center of the defining aspects of society.… it is this slight difference between like how the world feels okay and then it doesn’t. And it is not this very complicated change. Being married is sort of like that, it is not that it changed things radically, but there is this little piece of…You are a part of something, you know?*

(Ludo)

#### 3.2.4. The Hybrid Toolbox

All participants described a multitude of strategies from the different toolboxes that they employed to help them live through their often-frequent desire to die, because as they had come to learn, there was no one magic solution.

*You should have something that is meaningful, you should exercise, so I try to do all those things because no one thing will (help)… It is not that I will come to understand all my underlying crap, and I will, you know, let go of that stuff, or I will put it in a different place, which is usually how I think about that stuff because I don’t want to just get rid of it, because I don’t think you can get rid of your history … so I think for me, I just have to be really committed to doing all these different things at once.*

(Ludo)

Most participants in this study had experienced suicidal behavior over a long period of time, often since their childhood. This meant that they expected to be managing it for the rest of their lives, but also that they had had a long time to build an effective hybrid toolbox. That toolbox was not static, and a tool that may have once worked often became obsolete for a time or during a particular stage in the journey of suicidal behavior. However, participants knew they may draw on it again in the future, even if it was at the back of their toolbox for now. Equally, they would find other tools as their life and circumstances changed and evolved.

*It (suicidal behavior) has been with me my entire life and it was the only thing I took comfort in for a long time, for years and years… it was my only comforting thought. I know it will be again … one thing I have been practicing in my good times is every day, like I said, meditation… and just sitting down and remembering all the things that are good in my life, and it is very important to stay grateful, like, I have an apartment, it is great, and I can afford it, thanks to my disability. You know, my neighbors are some of my best friends in the world and any time I need them I can go down, and any time they need me they can go up. I love my job. I have never been able to say that before and I have had a lot of jobs. I love my job.*

(Alan)

## 4. Discussion

This study has explored the lived experience of dynamic stages of suicidal behavior and the unique toolboxes that people with such experiences develop to live with and manage their suicidal behavior. Our aim in utilizing these firsthand accounts of living with and through suicidal behavior was to examine what we could learn from participants’ experiences to enhance suicide prevention efforts.

While it is often stressed that the lifetime risk of suicide for people who have engaged in suicidal behavior heightens as they age due to practice [6,20], participants in this study demonstrated that they also became very skilled and competent at not only managing but growing through ongoing suicidal behavior. This fits with the concept of post-traumatic growth (PTG), whereby individuals who have struggled with a devastating event or series of events grow in positive ways. These personal changes imply that they do not merely survive and return to how they were prior to the event, but rather they grow in profound ways and reach a new sense of meaning and wisdom in their lives [21]. 

In line with other findings in the field of PTG [22], participants in this study reported having experienced growth across all five domains of PTG: increased appreciation for life, increased importance of relationships with others, finding new opportunities, personal strength, and spiritual growth [22]. Perhaps it is not surprising that most participants in this sample experienced post-traumatic growth, given that the two key personality characteristics that are proposed as necessary—extraversion and openness to experience [21]—would make participants with those qualities more likely to participate in research and share their story. Nevertheless, future studies can enhance our understanding of the phenomenon of PTG in individuals who have survived or are living with suicidal behavior [22,23]. 

Cognitive processing or ‘grief work’ and self-disclosure are described as central to PTG [21] and most participants in this study described storytelling—both telling one’s own story and hearing the stories of others—as a key tool for healing. The term storytelling, used by several participants, implicitly describes self-disclosure with more depth. Previous research on the benefits of self-disclosure after a suicide attempt has demonstrated that disclosing one’s experience of suicide in more depth leads to greater PTG [23]. In the same line, participants in the present study found disclosing to family, friends and health professionals to be largely beneficial, because disclosure provided the opportunity for support, a recurrent finding in the literature [23,24].

Intriguingly, however, our study found that storytelling as a helpful tool was discussed most frequently in the context of formal and informal peer-support. Storytelling included both telling one’s own story of suicidal behavior and finding catharsis and support, as well as hearing others’ stories and finding hope and learning. Telling and hearing stories of suicidal behavior also provides opportunities for rumination, which has been found to be crucial for PTG as it allows for cognitive processing and a positive reassessment of events [20,22]. Participants in this study were clearly adept at disclosing their experience of suicidal behavior, given that they mostly volunteered the information very early on in the interview. Many of them said that they had told their story numerous times over the years, as they liked to share it to help others, which in turn provided them with the opportunity for reflection [23].

As noted in the results, four of these participants reported volunteering or working in formal peer-support, in addition to most having utilized either formal or informal peer-support. The research on the effectiveness of peer-support for suicide prevention is surprisingly limited, given the increasing popularity of peer-support [25]. A systematic scoping review by Schlichthorst et al. (2020) [25] yielded seven records of eight suicide prevention peer-support programs, and only three of those had evaluations. The authors of the review concluded that, while peer-support programs for suicide prevention show promise, there needs to be better evaluation [25]. The results of this study, while limited, indicate that peer-support can be beneficial for suicide prevention, particularly in relation to shared stories.

Relevant research on the risks and benefits of self-disclosure of suicidal behavior indicate that caution is needed, however, when sharing such stories [26]. While many of the benefits have been discussed in this paper, some of the risks include stigma, discrimination, unwanted psychiatric treatment, unsupportive reactions, loss of privacy, and emotional difficulties related to sharing or hearing such stories [26]. In addition, stories of suicidal behavior that contain explicit details of suicide or self-harm methods can be potentially risky in terms of triggering a copycat effect [27]. However, most work in this area has largely focused on media reporting of suicide as problematic in relation to stories of suicide; by contrast, little is known about any potential risks associated with sharing stories via peer-support contexts. Suicide-prevention initiatives such as ‘Live Through This’ [28], an online archive of intimate stories of suicide attempt survivors as shared by themselves, are growing in popularity, with the purpose of helping others who are managing suicidal behavior. In addition, organizations such as Changing Minds provide resources on how to safely share personal stories about suicidal behavior and mental health, educating the public in ways that are aimed to protect both the storyteller and the audience [29,30,31].

When sharing their stories, participants in this paper frequently discussed their reasons for living (RFL), a construct originated by Marsha Linehan, who is a survivor of suicidal behavior herself [18]. The RFL Scale developed by Linehan asked subjects to choose reasons for living on a checklist, when they might otherwise choose suicide [18]. In the original paper by Linehan et al. (1983), six primary reasons for living were indicated: Survival and Coping Beliefs, Responsibility to Family, Child-Related Concerns, Fear of Suicide, Fear of Social Disapproval, and Moral Objections [18]. 

The RFL Scale has undergone systematic review and was found to be useful in moderating suicide risk [32]. While ‘fear of suicide’ and ‘fear of social disapproval’ [18] did not come through in these participants’ narratives as reasons for living, moral objections (such as spiritual beliefs), child-related concerns, responsibility to family, and many of the survival and coping beliefs in RFL formed a part of these participants’ toolboxes [18].

Additional tools described by participants in this study, and ones not in the RFL inventory, included broader positive social and policy changes (e.g., same-sex marriage rights and anti-stigma campaigns, bridge barriers as demonstrating community care), the ability to reinvent oneself, exercise, nature, storytelling, peer-support, and pets. Many—though not all—of the tools or reasons for living to emerge from the present analysis were also found in a recent study by Hawgood et al. [33] (pets, broad social policy and societal change did not appear in that investigation). In addition, while Hawgood’s study found that being oriented toward the future helped people live [33]—and this was the case for many participants in the present study as well, especially for young people—other participants found that being focused on the present was most useful.

Vatne et al. (2016) also examined what resources strengthened patients’ desire to live after a suicide attempt [34] and found that a sense of connectedness and having someone who cares was crucial, findings that resonate with this study. Vatne et al. [34] additionally described a key theme ‘becoming aware of the desire to live’, which was essentially a state of ambivalence for patients in their study, described by the authors as “as a struggle in which hope is strengthened at the same time as longing to escape something unbearable is present” (p. 10) [34]. Bergman et al. (2017) also explored the state of ambivalence in patients who had survived suicide attempts, albeit repeated suicide attempts (RSA) [35]. The description that Bergman and colleagues gave to the state of ambivalence for patients with RSA is quite different to that of Vatne et al. [34,35], however, as well as that offered in this article. This may be because Bergman et. al.’s study [35] focused on RSA (although several participants in our study did make RSA, with some estimating that they had made anywhere between four and 12 suicide attempts). Bergman et al. [35] describe the “torment” (p. 633, 646) for participants in their study when living in a state of ambivalence, due to the ambiguity and complexity of making decisions that are literally life and death. However, Bergman et al. [35] also describe the state of ambivalence as providing a “lifeline” (p. 638) as they could decide to live while still keeping the possibility of death as an option. Participants in our study, by contrast, did not describe the state of ambivalence as torment [35], but rather a feeling of not being overly attached to being alive, nor being currently suicidal. Some described this state as even ‘romantic’ or like ‘a game’, whereby their suicide attempts, while ambivalent, were not serious (but potentially lethal nonetheless). As such, this state is no more risky than other suicidal states.

As with Bergman et. al.’s study [35], the possibility of suicide was comforting to many participants in our study, and perhaps counter-intuitively, this acknowledgement did help them to live through difficult times. Knowing that they at least had autonomy over their own life and could choose suicide helped them to survive, at least in the moment.

The torment state, described in Bergman et. al.’s [35] study was arguably more consistent with the fighting for life stage in our study, where participants described feeling exhausted by the constant daily battle with managing suicidal behavior. During this stage of suicidal behavior, participants wanted to be protected from themselves, which is consistent with other research exploring the experiences of those who have survived suicide attempts [36]. Berglund et. al.’s research [36] also found that patients in their study sought autonomy, connections, and talking, to regain or find hope in their lives after a suicide attempt. Finding or holding hope was a key stage in the experience of managing suicidal behavior for many of the participants in our study—though not all—and the sources of hope were consciously developed through their unique toolbox. As with Berglund et al’s [36] research findings, talking (i.e., storytelling), seeking connection, and having autonomy were important tools for many participants in our study.

Maple et al. (2019) [37] also reported on the experiences of suicide attempt survivors, and as with our study, participants were not purposely selected as suicide attempt survivors, but instead self-disclosed. The authors [37] identified three themes in their analysis of the qualitative data in their study: the experience of being suicidal, talking—or not—about suicide, and mental health care systems [37]. Aspects of these themes echo our own findings; in both studies, participants described how it felt to be suicidal and the acceptance of learning to manage their suicidal behavior, rather than resolving it. In addition, the talking about it—or not—theme resonates with aspects of the storytelling tool in the self-directed toolbox, as participants in Maple et al.’s study also used their stories to help others [37]. Finally, the mental health care system theme, in which participants in that study discussed how treatment had often been life-saving, was also similar to points raised by our interviewees, with some individuals in both studies expressing a desire for a more holistic treatment. One key message to emerge from the present research was that while many participants found the mental health system helpful, they often found the most help from other patients and peer support.

## 5. Limitations

This qualitative study was based on self-reported data from 17 participants who had self-disclosed suicidal behavior. The study involved open-ended narrative interviews and participants were not consistently or systematically asked if they had personal experience of suicidal behavior. Hence, the findings are not necessarily representative of those with similar histories. As participants experienced different types of suicidal behavior simultaneously/at different points, it was not possible to analyze participants’ experiences based on specific types of suicidal ideation/behavior. Further studies, including quantitative studies, are needed to investigate, for example, coping strategies of people who have attempted suicide or are experiencing suicidal ideation.

## 6. Conclusions

This study provided first-hand insights into how people experienced their suicidal behavior and found ways to live with and through their desire to die by suicide. Their effective coping strategies often strayed beyond traditional mental health approaches. In general, this pattern of findings suggests a need to look beyond the medical model of suicide prevention and to adopt strengths-based frameworks in collaboration with those with lived experience of suicide. Empowering people who are suicidal can be achieved through helping them to identify their strengths and coping skills. Alongside proven interventions involving formal medical or therapeutic support, this process may involve working in partnership to encourage those at risk of suicidal behavior to generate their own solutions and coping strategies—an approach that is supported in best-practice suicide prevention safety planning [38,39,40].

Although suicide risk may sometimes increase over time [6,20], suicidal people can become adept at living skillfully with their suicidal behavior and can actively cultivate strategies to live well. This ‘living well’ can be further facilitated in suicide prevention efforts by working alongside people with personal experience of suicidal behavior, strengthening peer-support services and adding value to the storytelling process. Collaboration takes time and requires good relationship building. As Stefan [41] (p. 479) writes:

*As a society, we see preventing suicide as analogous to snatching a person out of the way of a speeding vehicle. We don’t see it as walking along the road with a person who is completely lost and uncertain about his or her destination, when we don’t know the destination either. It may be a long way away. The person may never get there. But the walk is the process; it’s how we stay alive.*

In sum, findings from the current study shed light on how suicide prevention efforts can more effectively support people to not merely live, but to live well. By taking the time to walk alongside someone on the journey of managing suicidal behavior, powerful relationships and tools can be formed that have the potential to save, and sustain, lives.

## Figures and Tables

**Table 1:** Sociodemographic characteristics of participants (*N* = 17).

Participant Characteristics	Number (*n*)
Age (years)	
18–24	5
25–44	7
45–64	4
65+	1
Sex/Gender	
Male	6
Female	9
Gender Diverse	2
Ethnicity	
White	16
Middle Eastern	1
Suicidal Behavior	
Attempted Suicide	11
Suicidal Ideation with Suicide Plan	5
Suicidal Ideation over a long period of time without Suicide Plan	1
LGBTQIA+	3
Religious/Spiritual Beliefs	15
Childhood Trauma/Abuse	14
Formal Peer Support Worker	4

**Table 2:** Description of themes.

Theme	Subtheme
1. Dynamic relationship with suicidal behavior: Living with, and through	Fighting for life Ambivalence Acceptance Finding and holding hope
2. The toolbox	The self-directed/discovered toolbox The conventional/medical toolbox The life circumstances toolbox The hybrid toolbox

## Data Availability

Due to the identifying nature of these qualitative interviews, full transcripts cannot be shared for ethical and privacy reasons.
